# Supplementary material for: SARS-COV-2 Omicron variants conformationally escape a rare quaternary antibody binding mode
Source: Commun Biol. 2023 Dec 11;6:1250. doi: 10.1038/s42003-023-05649-6 (PMC10713552; doi:10.1038/s42003-023-05649-6)
Supplement: Supplementary file 2 — Supplementary information [file 42003_2023_5649_MOESM2_ESM.pdf]

Supplementary Information for:

SARS-COV-2 Omicron variants conformationally escape a rare quaternary antibody binding mode

Authors: Jule Goike[1,2#], Ching-Lin Hsieh[2#], Andrew P. Horton[1,2#], Elizabeth C. Gardner[1,2#], Ling Zhou[2], Foteini Bartzoka[1,2], Nianshuang Wang[2], Kamyab Javanmardi[1,2], Andrew Herbert[8], Shawn Abbassi[8], Xuping Xie[14], Hongjie Xia[14], Pei-Yong Shi[14], Rebecca Renberg[4], Thomas Segall-Shapiro[3,9], Cynthia I. Terrace[3], Wesley Wu[9], Raghav Shroff[1,2,3,9], Michelle Byrom[1], Andrew D. Ellington[1,2,7], Edward M. Marcotte[1,2], James M. Musser[9], Suresh V. Kuchipudi[11], Vivek Kapur[12], George Georgiou[1,2,6,7,10], Scott C. Weaver[15], John M. Dye[8], Daniel R. Boutz[1,2,3*], Jason S. McLellan[2*], and Jimmy D. Gollihar[1,2,3,9*]

# Authors contributed equally
*Corresponding authors

Contents:

Supplementary Figures 1 to 16

Supplementary Tables 1 to 4

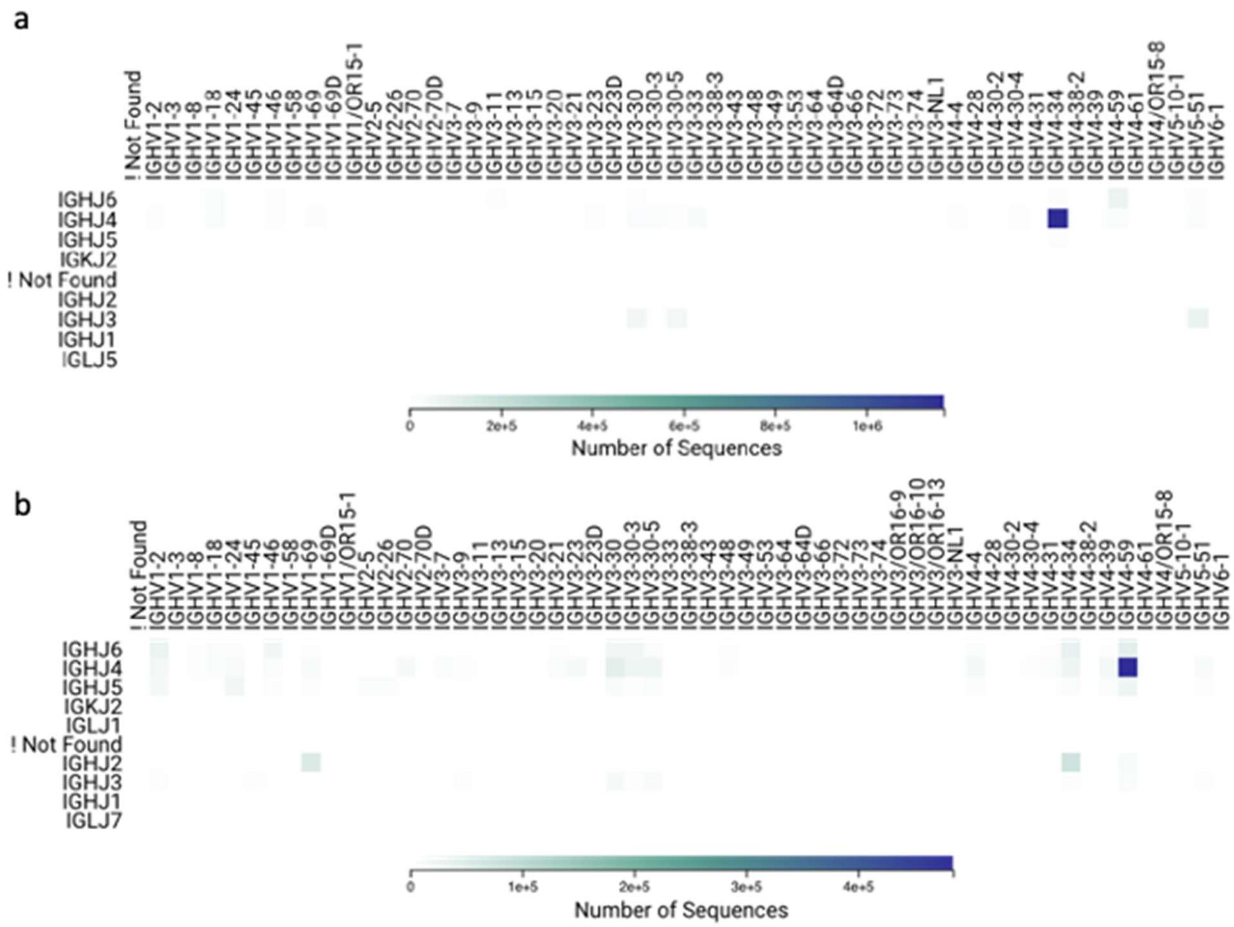

Supplementary Figure 1. Summary of IgG VH-only sequencing of donor 1 (a) and donor 2 (b). Libraries generated from these sequences were used for IgSeq proteomics and as a starting point for YSD selections.

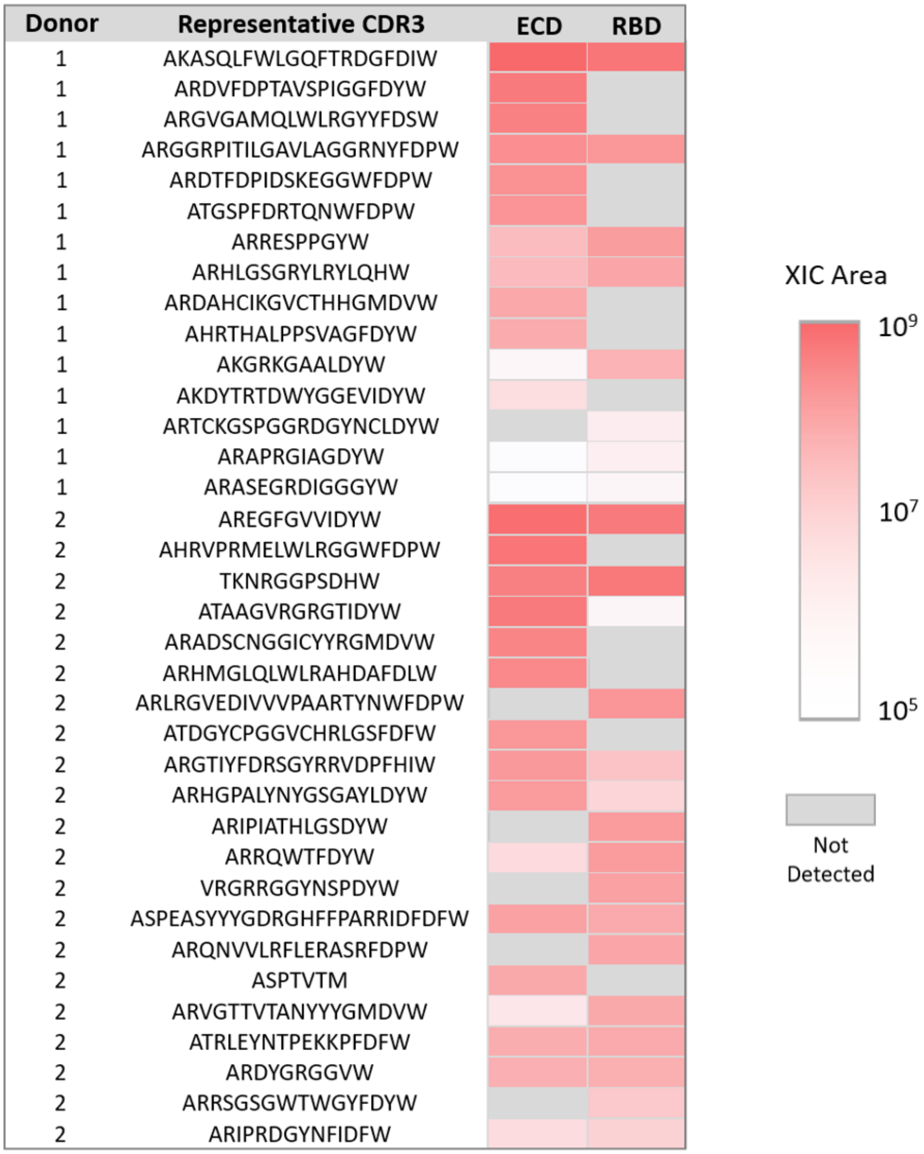

Supplementary Figure 2. Anti-SARS-CoV-2 ECD and RBD antibody clonotypes identified in the serum of donors 1 and 2 by IgSeq proteomic analysis. Heat maps represent the relative abundances of unique clonotypes calculated as the sum of XIC peak areas of CDR3-peptides observed by LC-MS/MS.

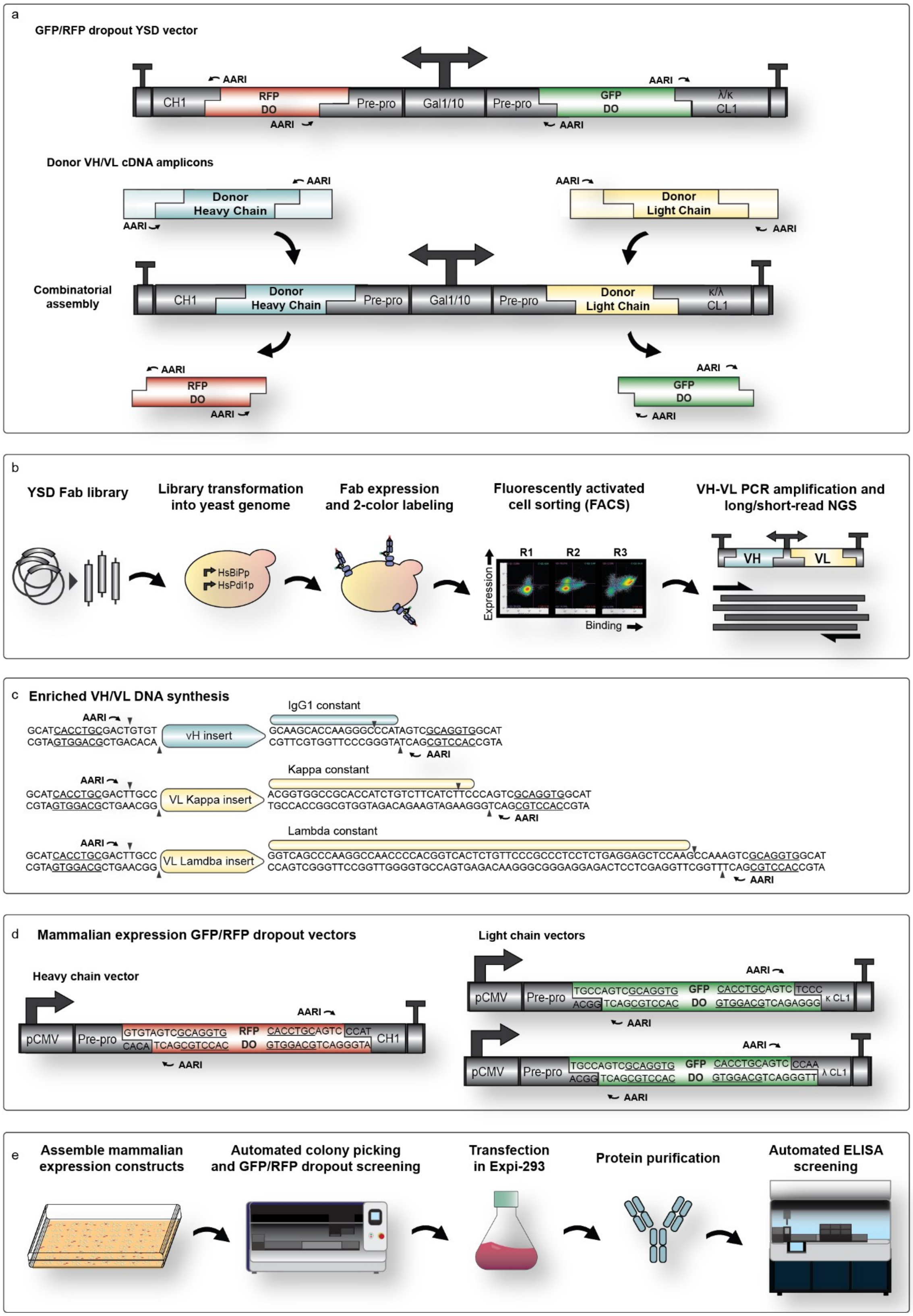

Supplementary Figure 3. Yeast Surface Display platform. a, Donor heavy and light chain cDNAs were combinatorially assembled into a GFP/RFP dropout Golden Gate vector. **b**, Libraries were integrated into humanized yeast strains optimized for surface display. Yeast libraries were sorted via FACS and subject to short and long read sequencing. **c**, Enriched antibodies determined by next-gen sequencing were synthesized as e-blocks (Integrated DNA Technologies) or PCRed directly from yeast genomes. **d**, VH or VL inserts were cloned into mammalian GFP/RFP dropout Golden Gate vectors. **e**, Cloned mAbs were drop-plated for automated colony picking in a QPix 420 (Molecular Devices). Verified mAbs were transfected, purified, and subject to a high-throughput ELISA assay programmed on a Tecan Fluent 1080.

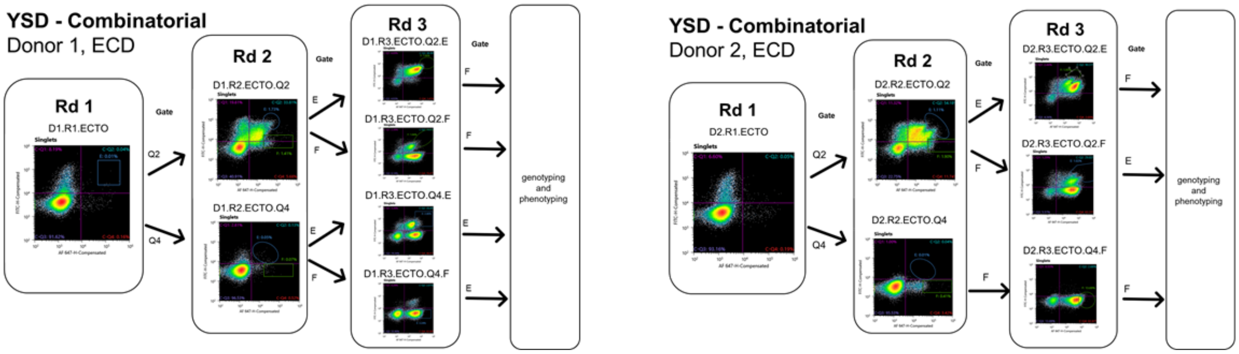

Supplementary Figure 4. Representative YSD cell sorting lineage plots. Donor repertoires were cloned as Fab libraries and displayed in yeast. Yeast were labeled for expression (y-axis, anti-FLAG-FITC) and antigen binding (x-axis, biotinylated ECD, Streptavidin-Alexa Fluor 488; human-Fc RBD, anti-human Alexa Fluor 488). Each library was subjected to selection in the presence of either RBD or spike ECD. Each population was subjected to sorting into up to two gates at a time, such that after several rounds of selection, populations with various expression and binding characteristics were enriched (see Rd 3). Combinatorial libraries consisted of randomly-paired VHs and VLs cloned from donor cDNA.

a

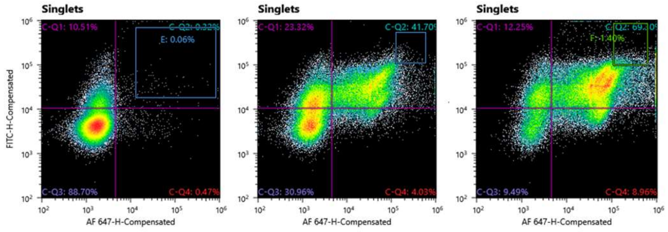

b

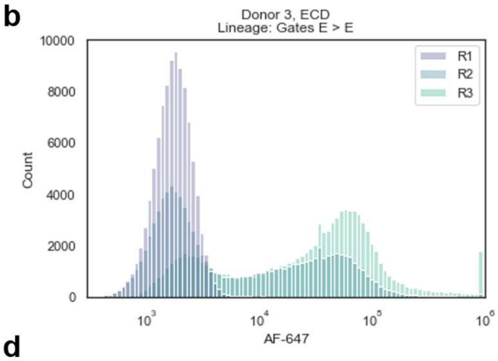

c

Rank	CDRH3	Enrichment factor	Rank by read count	Change
1	ATASPHCSSTSCSINWFDP	9988	2	1
2	ASGGGSYYSPFDY	2413	7	5
3	ARAGGGGYQGPFDY	1991	1	-2
4	ARAYGGGYLTPFDY	1391	4	0
5	AKPQGGGYNGAFDI	720	6	1
6	ATVSPFWSGYYNNWFDP	407	3	-3
7	ARPVTGGYYYYMDV	380	8	1
8	TTERYYDYIWGSYRYSDY	342	10	2
9	ARDLRGSYSGLLDY	307	11	2
**10**	**ATRFAVYGDYLIDY**	286	9	-1
11	AKVVRHLYSSGYSNWFDP	233	20	9
12	ANQVTYYDYIWGSYRPGAFDI	150	13	1
13	ARDKGRGILFTHRDAFDI	140	29	16
14	ARRRVYPSGFDY	138	18	4
15	ARASHGGYSSAFDP	131	15	0
16	VRSGHCNGGVCYGAEYFQH	112	31	15
17	ARDGSGGYSNPDY	111	12	-5
18	VKGAGGYFDS	110	32	14
19	ATAPAVVVGNWFDP	94	34	15
20	ARGLDIIVVPAALDH	92	5	-15

d

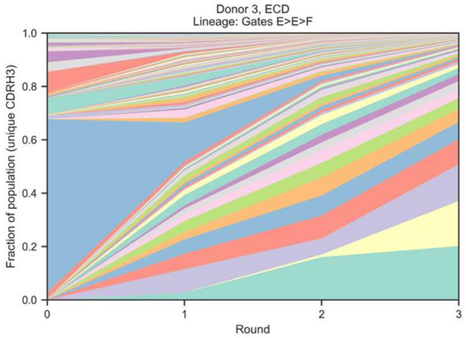

Supplementary Figure 5. Yeast surface display of a combinatorially assembled donor repertoire. a, Representative example of YSD cell sorting showing donor 3 Fab selection with the ECD antigen. The x-axis (AF-647) shows antigen binding and the y-axis (FITC) shows Fab expression level. Each round was sorted using one or two gates to enrich populations with different phenotypes. **b**, Histogram showing enrichment of binders from round one (purple) to round 3 (light green) in the highlighted lineage. **c**, Table showing the top HCDR3s as ranked by enrichment in this lineage. Relative ranking by raw readcount is also compared. The bolded HCDR3 represents neutralizing mAb 7-6. **d**, Area chart showing HCDR3 enrichment throughout selection. Each unique HCDR3 is represented as a fraction of all HCDR3s in that population. Significant bias exists in the initial library, but by the end of the selection top variants represent over 10% of the total population.

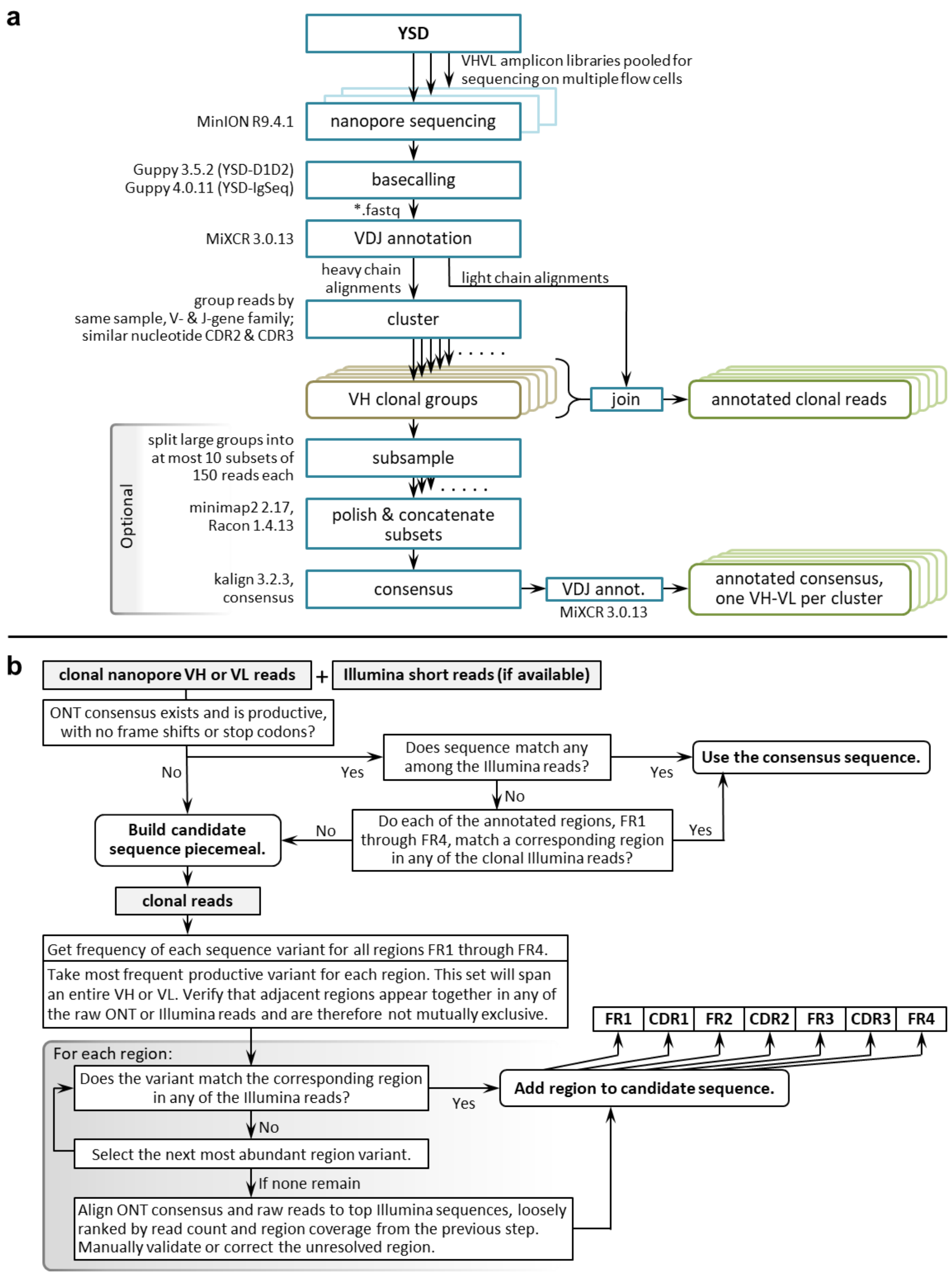

Supplementary Figure 6. Definition of candidate VH-VL sequences from error-prone MinION sequencing. Nanopore long read sequencing (ONT) enables readout of paired antibody heavy and light chains in a single read. **a**, Nanopore long read VH-VL sequencing, annotation and read clustering. Once reads are acquired and base called, we use MiXCR for antibody sequence annotation. This identifies heavy and light chain position in each read and the delineates the VH and VL framework (FR) and complementarity determining regions (CDR). Despite being developed for high quality sequencing, the MiXCR annotation function works quite well on nanopore (ONT) data. We then group reads through iterative stages of growing and splitting sequence clusters. Each final cluster, or clonal group, aims to capture all raw MinION reads that derive from a single canonical VH sequence, and due to sequencing error, a cluster often contains many thousands of unique reads. A clonal group may also include a consensus sequence obtained through sequence polishing and alignment. **b**, Antibody VH and VL sequence definition from clonal nanopore reads. When available, the ONT clonal group is supplemented with any sample-matched Illumina short reads that have an amino acid CDR3 matching one among the ONT group.

VH CDR3	MinION 1.7-2.1kb total reads	MinION all total reads	iSeq total reads
AKTSGYNLPDY	2467936	3218688	82347
ARGTIYFDRSGYRRVDPFHI	682539	1153588	41256
ATGPAVRRGSWFDP	189383	250994	14595
ARRQWTFDY	170557	198948	5961
ATGSPFDRTQNWFDP	150096	195000	9471
ARDGGGYVSY	137057	184361	7760
ARGVGAMQLWLRGYYFDS	101311	136418	10220
TTDRTYDYVWGSYRYEDY	18251	26192	1497
ASPEASYYYGDRGHFFPARRIDFDF	14123	19357	1356
TTDRSYDYVWGSYRYPDY	13957	20327	1046
ARIPIATHLGSDY	13507	20181	1058
ATAAAVRGRGTIDY	11135	17518	924
ARDYGRGGV	9303	12940	631
AKDRAIFYLNPRYYLDY	9245	12123	881
ASPTVT	8847	11590	436
ARELPPGRMVVPATYWHFDL	7591	11084	928
TSPRADYGSNSVGNY	5736	7607	725
ARDGITNSGVVTHFGMDV	4214	5713	320
ARHMGMQLWLRAHDAFDL	908	1137433	46761
ARLGTGYP	655	1077	32
ACSRARGFMFFDY	631	1850171	71835
AKATQLFWLGQFTRDGFDI	366	2110	128
ARADSCNGGICYYQGMDV	215	602990	27489
AREQPPGRMVVPATYWHFDL	70	8668	222

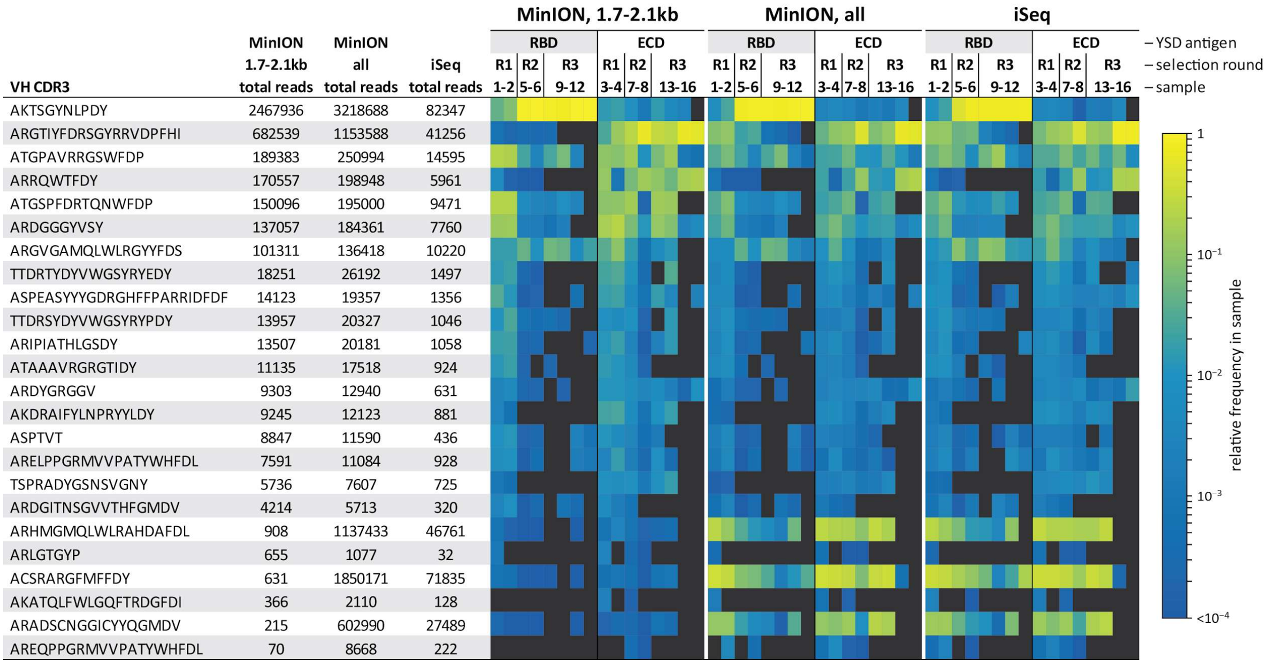

Supplementary Figure 7. VH CDR3 read counts and relative abundance from the IgSeq-YSD experiment, shown for MinION reads of 1.7 kb to 2.1 kb (left), all MinION reads (center) and all iSeq reads (right). Heatmap values are CDR3 frequencies, or read counts normalized within each respective sample. Length requirements improve VHVL abundance estimation by, in particular, removing the inflated counts of short artifactual reads due to unbalanced PCR amplification. Comparison with and without filtering shows certain CDR3 are disproportionately affected. The iSeq read counts for a VH CDR3 include only reads with identical amino acid CDR3 annotation. However, high MinION error rates, on the order of 1 error per 10 bases, create enormous sequence diversity, and the respective MinION count for each CDR3 includes all those recovered through sequence clustering. (center, right panels) There is strong correspondence between the unfiltered MinION and iSeq CDR3 frequencies. This indicates that our MinION read consolidation techniques successfully recapitulate CDR3 patterns observed from the much more accurate Illumina sequencing platform.

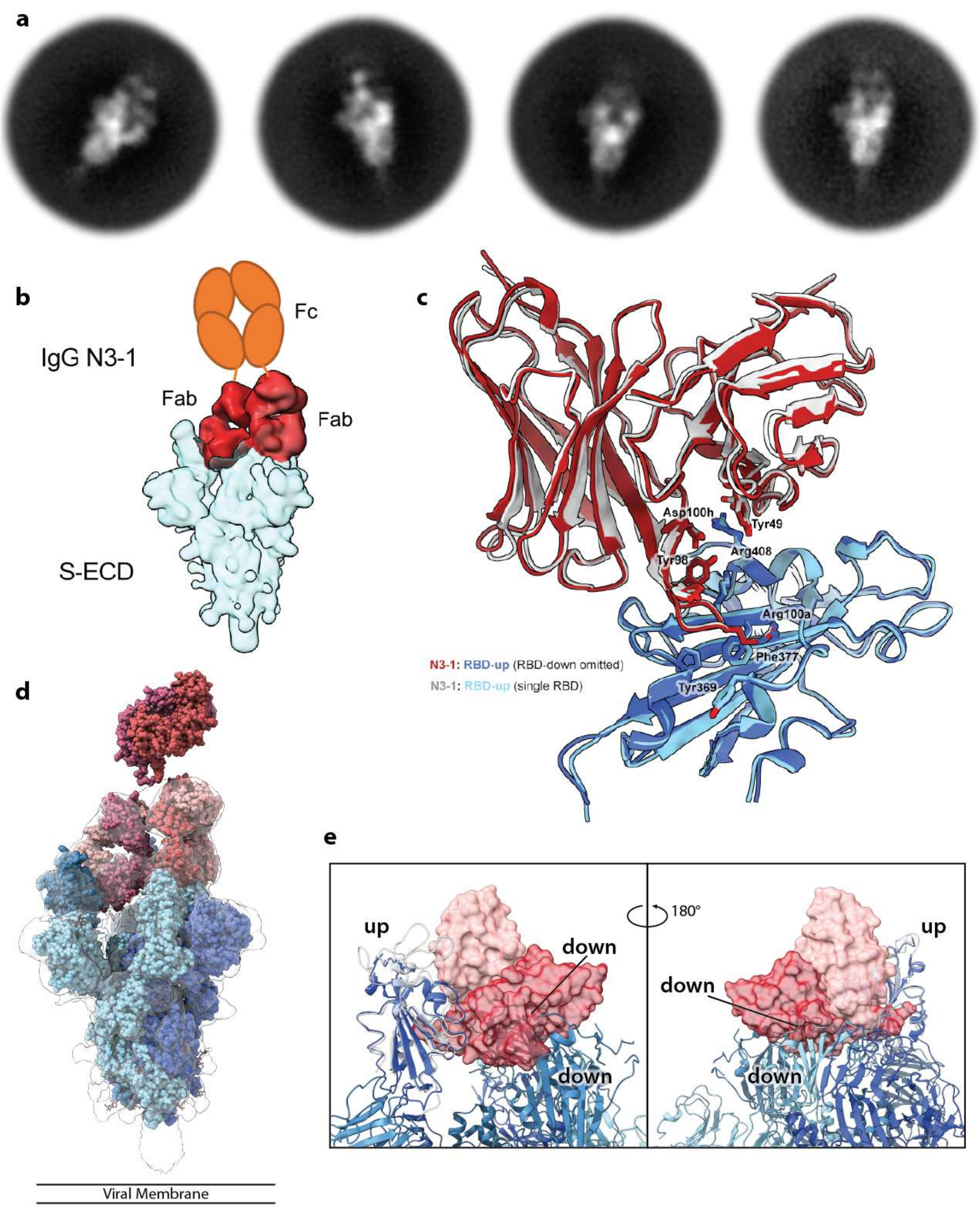

Supplementary Figure 8. **Avidity of mAb N3-1 likely achieved by a single IgG binding to a trimeric spike**. **a**, Representative 2D class averages of N3-1 IgG complexed with SARS-CoV-2 S by negative stain electron microscopy (nsEM). Although the density of the Fc is not well resolved, two clear densities of Fabs are visible per trimeric spike. **b**, A schematic model generated by Gaussian-smoothened cryo-EM map of N3-1 bound to SARS-CoV-2 S. The Fab density is highlighted in brick red, and the spike is shown in light blue. The unobserved Fc is shown as orange ovals. **c**, Superimposition of two focused maps of N3-1 bound to the single RBD up and the two RBDs in an up and down state. **d**, Modeling shows the N3-1 IgG can bind to spike trimer with two RBDs in the up conformation without clashes. **e**, Superimposition of N3-1 Fab bound to RBD structure from this study with apo spike structure that has two RBDs in the down conformation (PDB ID: 6XKL). Were it to bind 1 RBD-up, N3-1 Fab heavy chain would clash with both RBDs in the down conformation.

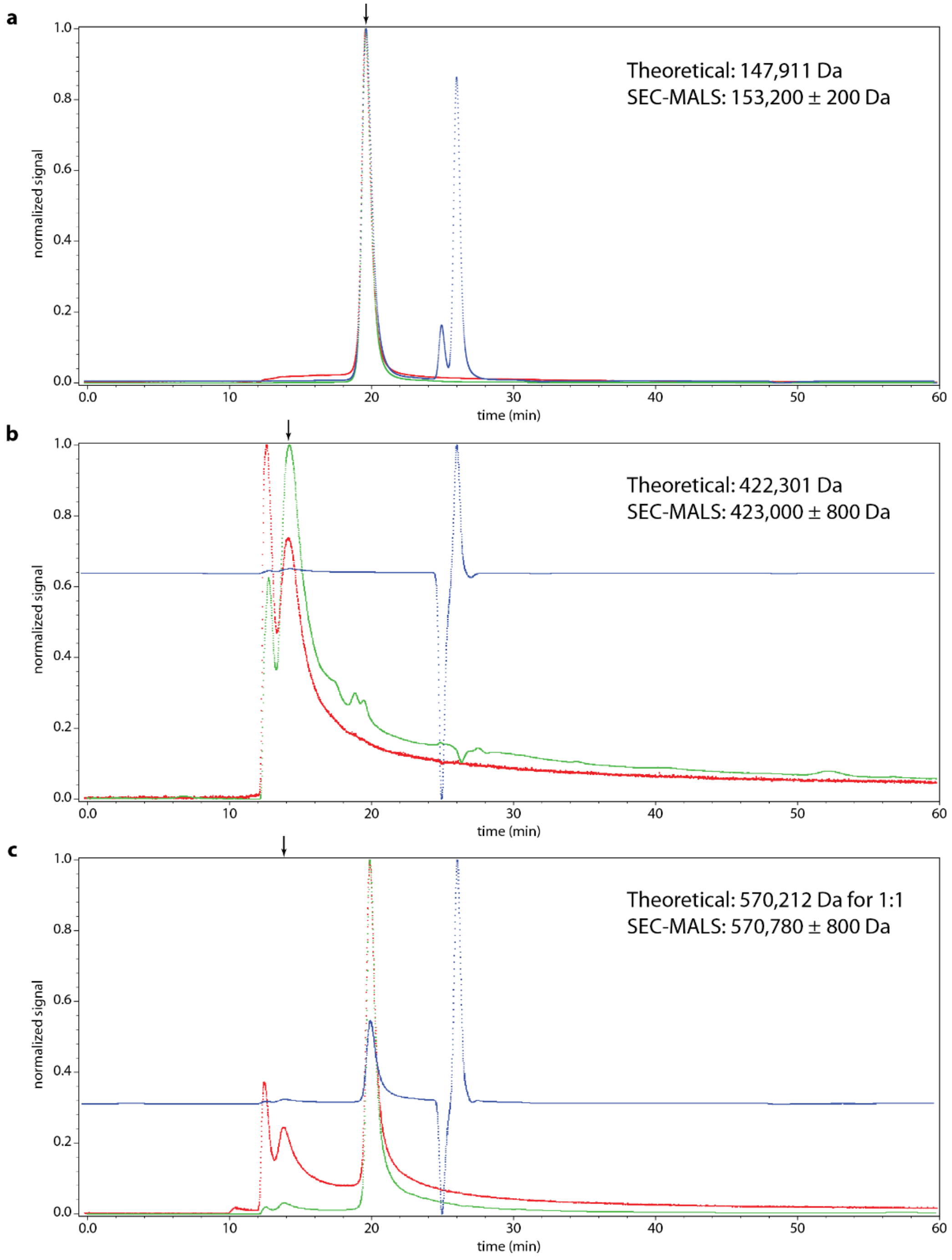

Supplementary Figure 9. SEC-MALS characterization of N3-1 and SARS-CoV-2 spike. a, SEC-MALS of N3-1 IgG shows a strong signal at 20 minutes, corresponding to the theoretical mass for the IgG. **b**, SARS-CoV-2 HexaPro spike peaks at 14.25 minutes, matching the theoretical mass of the spike trimer. The earlier peak indicates presence of high molecular weight spike aggregate. **c**, N3-1 IgG mixed with spike at a ratio of 3:1 yields a peak at nearly 14 minutes corresponding to a 1:1 IgG to spike complex. The second peak at 20 minutes shows an excess of unbound N3-1. SEC-MALS traces are normalized between 0 and 1 and display multi-angle light scattering (detector voltage, red), UV absorbance (AU, green), and differential refractive index (RIU, blue).

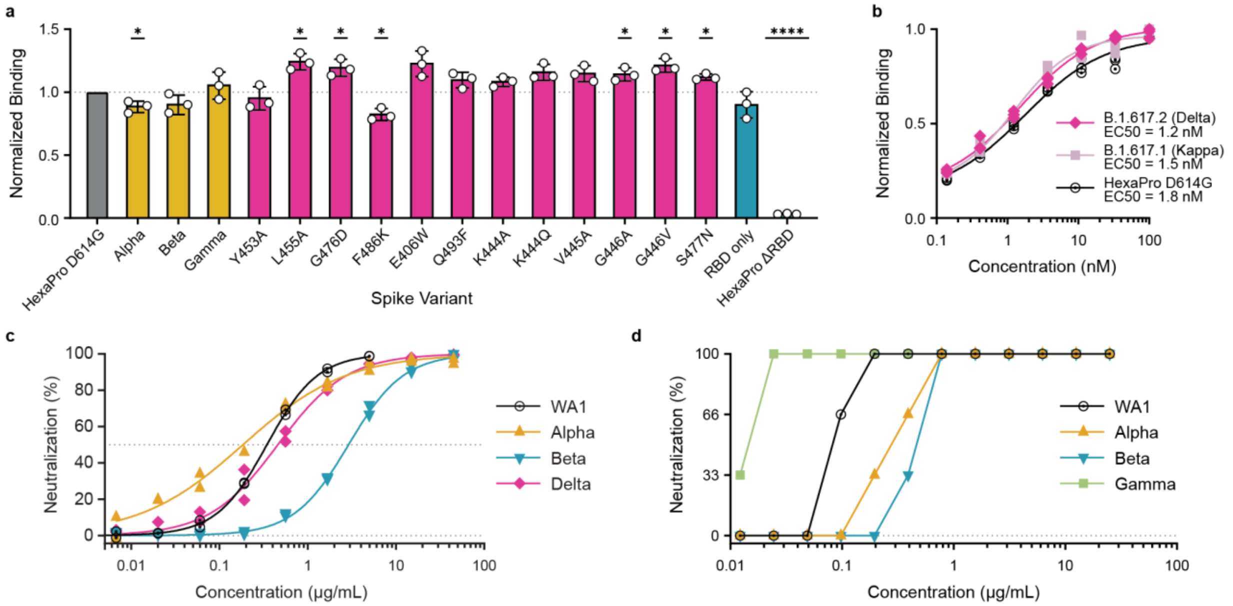

Supplementary Figure 10. RBD-directed mAb N3-1 neutralizes non-Omicron variants of concern. a, Spike variants were assayed against VOCs and Regeneron REGN10987 and/or REGN10933 escape mutants using mammalian surface display. HEK293T cells transiently expressing full length spike protein were stained with anti-spike antibodies and analyzed by flow cytometry. The median fluorescence intensity of the stained cells was normalized to the HexaPro-D614G spike. The SARS-CoV2 RBD subunit and SARS-CoV-2 spike with a deleted RBD (ΔRBD) were included as controls. **p*<0.05; *****p*<0.0001; using one sample t test (two tailed). **b**, Normalized binding of N3-1 to HexaPro and the Delta and Kappa variants of lineage B.1.617, assayed as in (**a**) but with multiple IgG concentrations to obtain dose response curves. **c, d**, Independent SARS-CoV-2 live virus neutralization assays for N3-1 IgG. Curves calculated from (b) three and (c) two biological replicates.

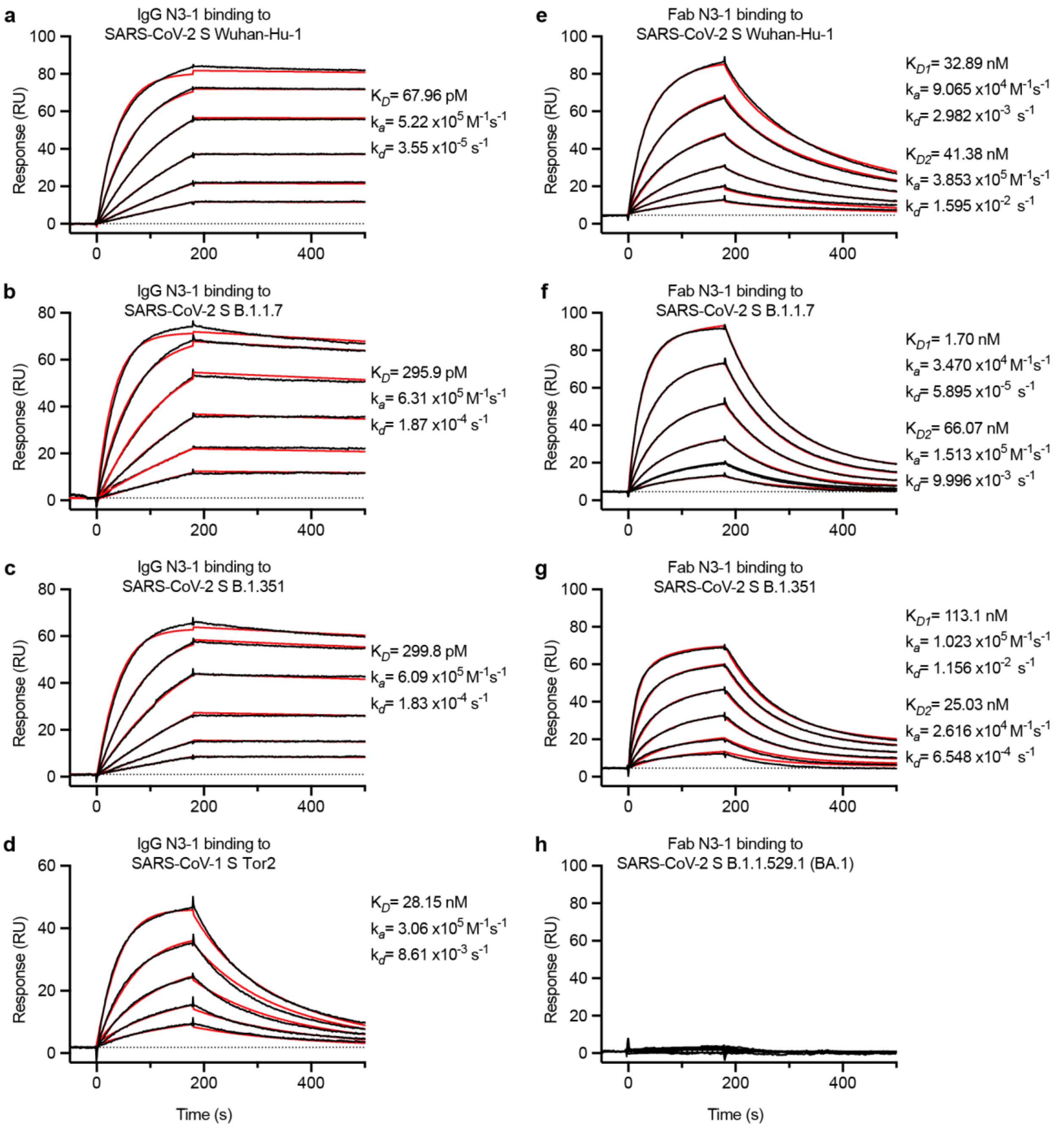

Supplementary Figure 11. **mAb N3-1 exhibits cross-reactivity and avidity to CoV spikes. a-c**, Binding of IgG N3-1 to SARS-CoV-2 S Wuhan-Hu-1 (a), its variants B.1.1.7 (b) and B.1.351 (c) were assessed by surface plasmon resonance (SPR) using an NTA sensor chip. **d**, Binding of IgG N3-1 to SARS-CoV-1 S was also assessed by SPR. **e-g**, Binding of Fab N3-1 to SARS-CoV-2 S Wuhan-Hu-1 (e), B.1.1.7 (f) and B.1.351 (g). Binding data are shown as black lines. For (a-d), the best fit was achieved using a 1:1 binding model and shown as red lines. For (e-g), the best fit to a heterogeneous binding model is shown as red lines.(h) No binding could be determined for Fab N3-1 to B.1.1.529.

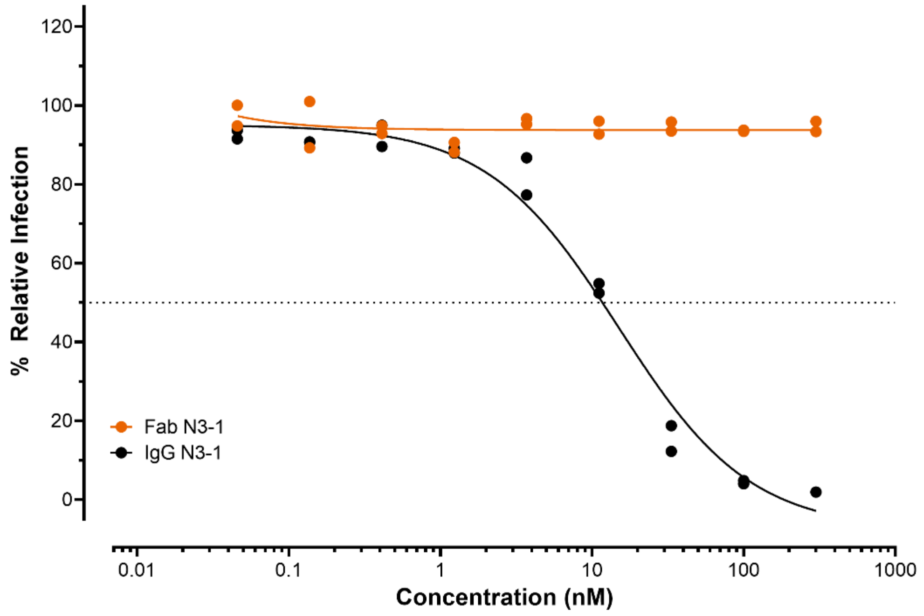

Supplementary Figure 12. Fab N3-1 does not neutralize WHU1. Live virus neutralization assay comparing the neutralization ability of IgG and Fab formatted N3-1 against the WHU1 SARS-CoV-2 strain. Curves are calculated from two biological replicates.

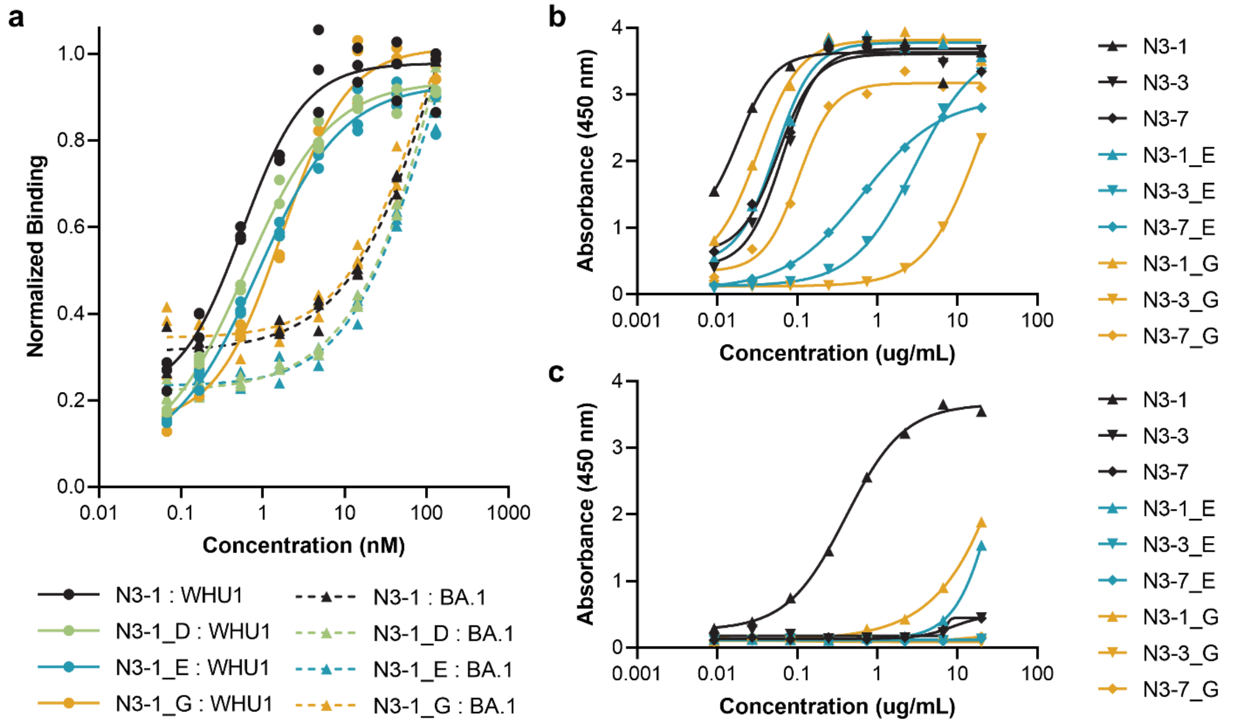

Supplementary Figure 13. Rationally designed N3-1 Thr57 variants do not improve binding ability to BA.1 spike. a, Normalized binding of N3-1 WT and N3-1 Thr57 variants D, E and G against WHU1 and BA.1 spike proteins in mammalian display assays. Curves calculated from three biological replicates. **b**, Binding of N3-1 Thr57 mutants paired with PLC1, PLC3 and PLC7 to BA.1 spike protein and **c**, WHU1 measured through ELISA.

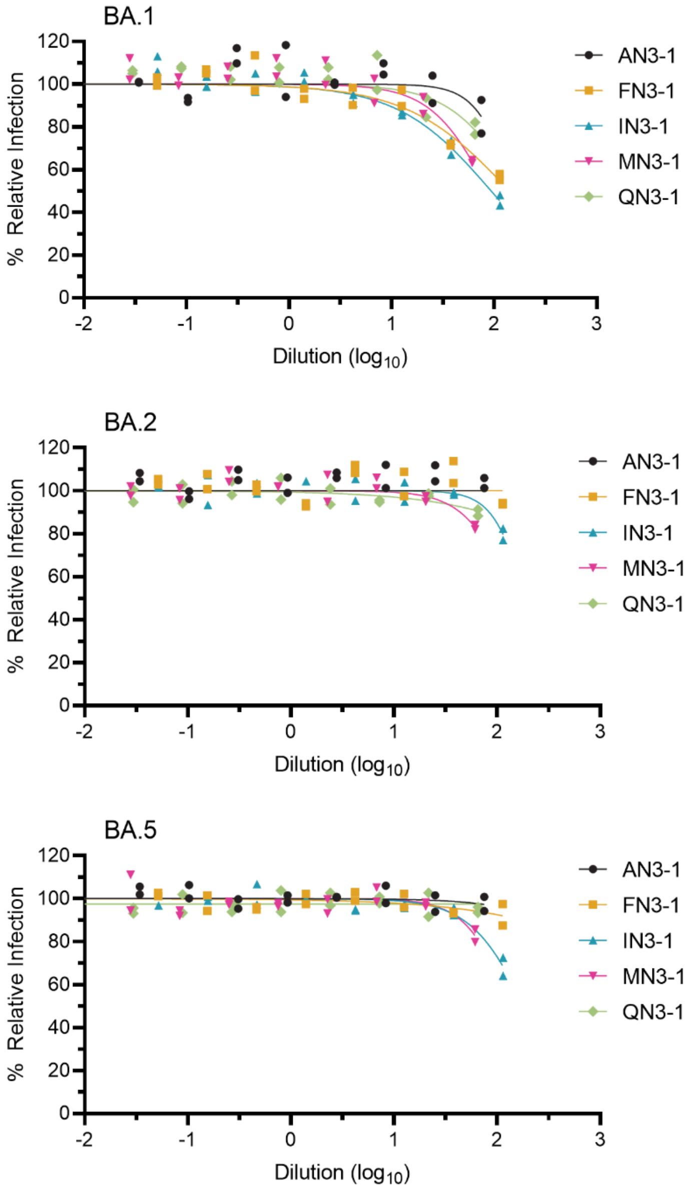

Supplementary Figure 14. N3-1 mutants show limited recovery of neutralization against Omicron variants. Live virus neutralization assay comparing the neutralization ability of IgG N3-1 mutants against Omicron variants BA.1, BA.2 and BA.5. While all N3-1 mutants have the poorest affinity to BA.1, BA.1 is still neutralized more effectively than BA.2 or BA.5. Plots show two biological replicates.

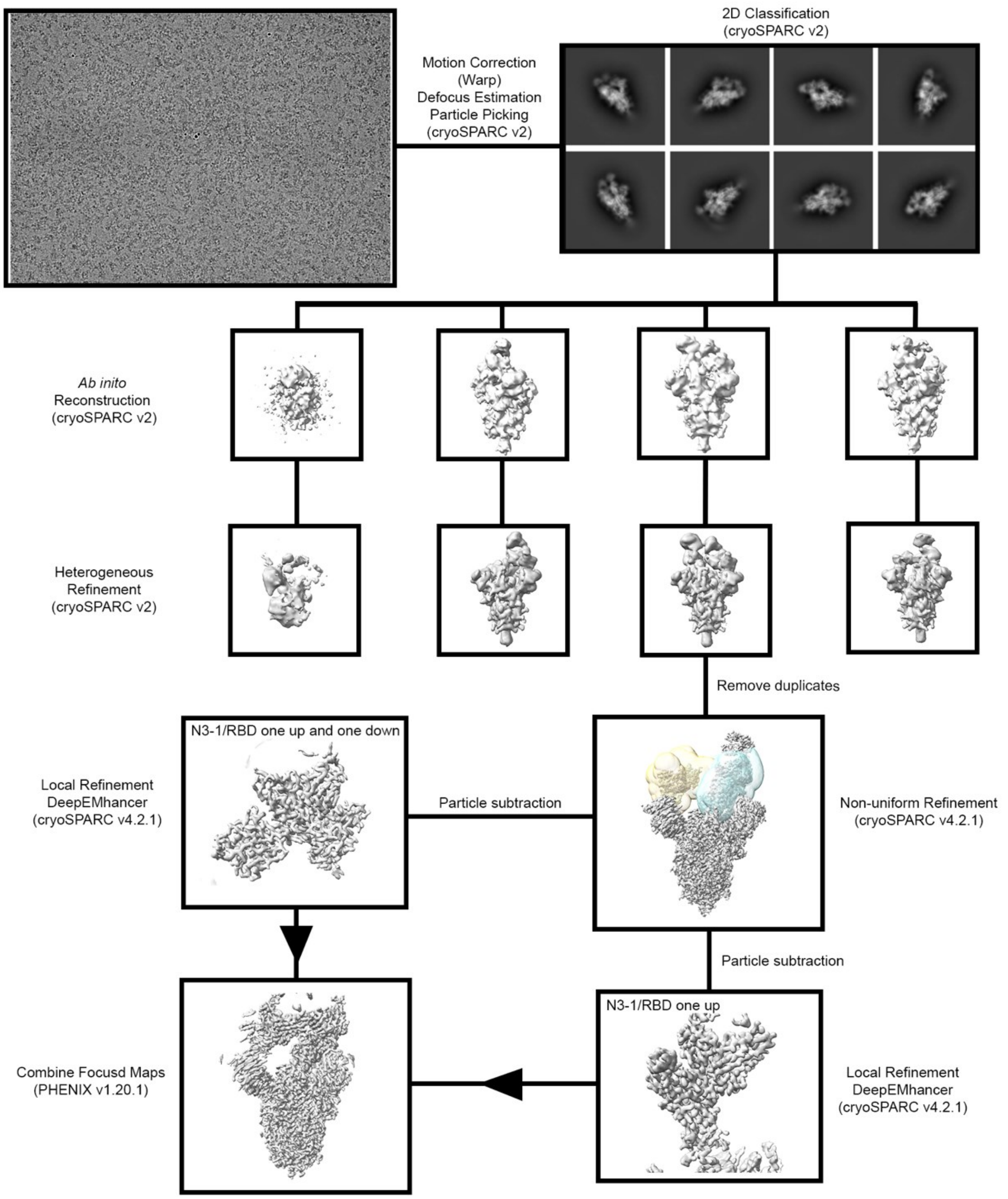

Supplementary Figure 15. Cryo-EM data processing workflow for N3-1 Fab bound to SARS-CoV-2 WHU1 S-HexaPro.

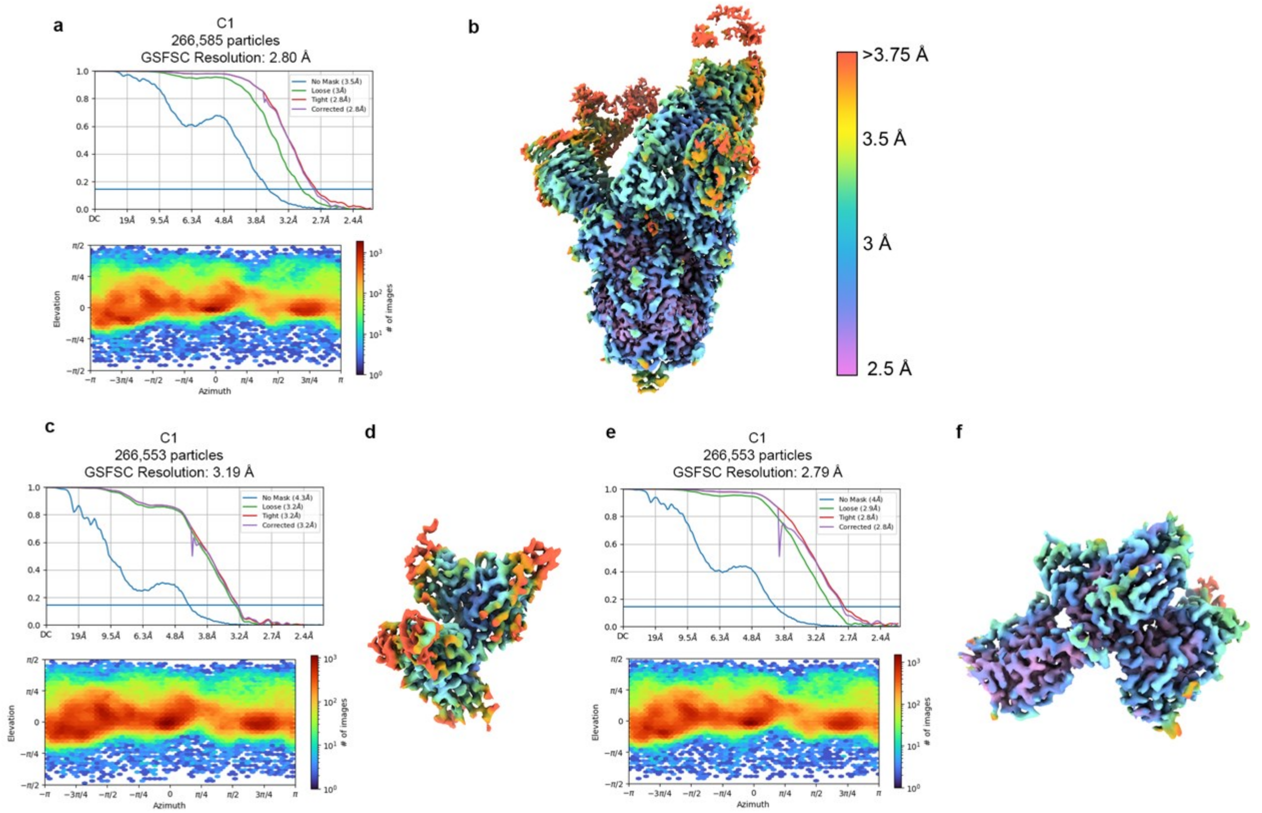

Supplementary Figure 16. Cryo-EM data validation. a,c,e, FSC curves (top) and the viewing direction distribution plots (bottom) for global reconstruction of N3-1 bound to SARS-CoV-2 S (a), focused reconstruction of N3-1 bound to one S-RBD (c) and focused reconstruction of N3-1 bound to two S-RBDs (e). **b,d,f**, cryo-EM map of N3-1 bound to SARS-CoV-2 S (b), one S-RBD (d) and two S-RBDs (f), respectively. The local resolution is depicted by a spectrum of rainbow color as defined in the key.

Supplementary Table 1. Summary of neutralizing antibodies

mAb	VH label	IC50 (nM)	VH gene	H-CDR3	VL gene	L-CDR3	Epitope	HC source	LC source
A7V32	A7	0.01806	IGHV1-24	ATGSPFDRTQNWFDP	IGLV2-8	SSYAGSNNLA	NTD	IgSeq	YSD
N3-1	N3	0.2519	IGHV4-31	ARGTIYFDRSGYRRVDPFHI	IGKV1-5	QQYNSYSPWT	RBD	YSD	PLC
A7V131	A7	0.3045	IGHV1-24	ATGSPFDRTQNWFDP	IGLV1-51	GTWDNSLSAGV	NTD	IgSeq	YSD
12C114	12C	0.3516	IGHV1-24	ATGPAVRRGSWFDP	IGLV1-51	GTWDSSLSGYV	NTD	IgSeq	YSD
12C8	12C	0.8355	IGHV1-24	ATGPAVRRGSWFDP	IGLV1-51	GTWDSSLSAVV	NTD	IgSeq	PLC
A7V3	A7	0.9547	IGHV1-24	ATGSPFDRTQNWFDP	IGLV1-51	GTWDSSLSAVV	NTD	IgSeq	YSD
A7V132	A7	1.391	IGHV1-24	ATGSPFDRTQNWFDP	IGLV1-51	GTWDSSLSAGV	NTD	IgSeq	YSD
12C115	12C	1.929	IGHV1-24	ATGPAVRRGSWFDP	IGLV1-40	QSYDSSLSGWV	NTD	IgSeq	YSD
4C7	4C	2.147	IGHV1-24	ATAAAVRGRGTIDY	IGLV1-44	AAWDDSLNGPVV	NTD	IgSeq	PLC
4C8	4C	2.353	IGHV1-24	ATAAAVRGRGTIDY	IGLV1-51	GTWDSSLSAVV	NTD	IgSeq	PLC
8G91	8G	2.586	IGHV1-69	AREQPPGRMVVPATYWHFDL	IGKV1-39	QQSYSTPYT	RBD	IgSeq	YSD
A7V8	A7	4.243	IGHV1-24	ATGSPFDRTQNWFDP	IGLV1-51	GTWDSSLSAVV	NTD	IgSeq	PLC
8H97	8H	4.955	IGHV3-30-3	ARGNWGSYYYGMDV	IGLV3-1	QSSDDSNQLV	ECD	YSD	YSD
8A3	8A	6.422	IGHV3-66	ARGGVVDTYYYYGMDV	IGLV7-46	LLSQSGAWV	NTD	IgSeq	YSD
8F90	8F	11.56	IGHV3-11	ARDGITNSGVVTHFGMDV	IGKV4-1	QQYNSYSRVT	NTD	IgSeq	YSD
N3-3	N3	13.09	IGHV4-31	ARGTIYFDRSGYRRVDPFHI	IGKV3-11	QQRSNWPPLT	RBD	YSD	PLC
8C100	8C	20.45	IGHV3-30	AKDRRYYDFWSGYVGSPYYYYYYGMDV	IGLV3-1	QSYGNNQGV	ECD	YSD	YSD
1D4	1D	25.49	IGHV2-70	ARIPIATHLGSDY	IGKV3-15	QQYNNWPPWT	RBD	IgSeq	PLC
N3-7	N3	27.66	IGHV4-31	ARGTIYFDRSGYRRVDPFHI	IGLV1-44	AAWDDSLNGPVV	RBD	YSD	PLC
N3-63A	N3	32.01	IGHV4-31	ARGTIYFDRSGYRRVDPFHI	IGLV8-61	TLYMGGGLLV	RBD	YSD	YSD
P4D96	P4	33.54	IGHV3-30	AKAPGQWLRFHYYGMDV	IGLV1-40	NSRDINSNHVL	NTD	YSD	YSD
8B5	8B	34.84	IGHV1-2	ARELPPGRMVVPATYWHFDL	IGKV3-20	QQYGSSPPWT	RBD	IgSeq	PLC
3B9	3B	51.36	IGHV3-30	ARDGGGYVSY	IGLV3-1	QAWDSSTVV	NTD	IgSeq	PLC
12C130	12C	65.13	IGHV3-30	ATGPAVRRGSWFDP	IGKV1-39	QQSYSTPRT	NTD	IgSeq	YSD
4A5	4A	65.72	IGHV3-30-3	AKASQLFWLGQFTRDGFDI	IGKV3-20	QQYGSSPPWT	RBD	IgSeq	PLC
N3-63B	N3	81.5	IGHV4-31	ARGTIYFDRSGYRRVDPFHI	IGLV1-40	QSYDGSLNDDVI	RBD	YSD	YSD
8J127	8J	113	IGHV3-30	AKATQLFWLGQFTRDGFDI	IGLV1-40	QSYDSSLSGWV	RBD	IgSeq	YSD
8K42	8K	124.2	IGHV3-30	ARDYGRGGV	IGKV1-39	QQSYSTRPLT	NTD	IgSeq	YSD
8D107	8D	126.9	IGHV3-30	AKTSGYNLPDY	IGKV1-39	QQSYSTPRT	ECD	IgSeq	YSD
1D1	1D	172.6	IGHV2-70	ARIPIATHLGSDY	IGKV1-5	QQYNSYSPWT	RBD	IgSeq	PLC
1D9	1D	182.5	IGHV2-70	ARIPIATHLGSDY	IGLV3-1	QAWDSSTVV	RBD	IgSeq	PLC
P4D3	P4	242.4	IGHV3-30	AKAPGQWLRFHYYGMDV	IGLV1-40	QSYGNNQGV	NTD	YSD	YSD
4A7	4A	>300	IGHV3-30-3	AKASQLFWLGQFTRDGFDI	IGLV1-44	AAWDDSLNGPVV	RBD	IgSeq	PLC
8I122	8I	>300	IGHV3-30	AKDRAIFYLNPRYYLDY	IGKV1-5	QQYNSYPLT	ECD	IgSeq	YSD
Q318	Q3	>300	IGHV3-30	AKQAGAYCSGGSCYSSSEADY	IGLV1-40	QSYGNNQGV	RBD	YSD	YSD
N3-63C	N3	>300	IGHV4-31	ARGTIYFDRSGYRRVDPFHI	IGLV3-1	QAWDSSTVV	RBD	YSD	YSD
N3-819	N3	>300	IGHV4-31	ARGTIYFDRSGYRRVDPFHI	IGKV1-39	QQSYSTRPLT	RBD	YSD	YSD
1D5	1D	>300	IGHV2-70	ARIPIATHLGSDY	IGKV3-20	QQYGSSPPWT	RBD	IgSeq	PLC
9C-118	9C	>300	IGHV3-30	ASPTVT	IGKV1-39	QQSYSSSWT	ECD	IgSeq	YSD
12C112	12C	>300	IGHV1-24	ATGPAVRRGSWFDP	IGLV1-40	QSFDSSLSGPVV	NTD	IgSeq	YSD
12C113	12C	>300	IGHV1-24	ATGPAVRRGSWFDP	IGKV1-39	QQSYSTRPLT	NTD	IgSeq	YSD

Name	V-Gene	J-Gene	Light CDR1	Light CDR2	Light CDR3
**PLC1**	IGKV1-5	IGKJ1	QSISSW	DAS	QQYNSYSPWT
**PLC2**	IGKV2-28	IGKJ2	QSLLHSNGYNY	LGS	MQALQTPPYT
**PLC3**	IGKV3-11	IGKJ4	QSVSSY	DAS	QQRSNWPPLT
**PLC4**	IGKV3-15	IGKJ1	QSVSSN	GAS	QQYNNWPPWT
**PLC5**	IGKV3-20	IGKJ1	QSVSSSY	GAS	QQYGSSPPWT
**PLC6**	IGKV4-1	IGKJ2	QSVLYSSNNKNY	WAS	QQYYSTPPYT
**PLC7**	IGLV1-44	IGLJ3	SSNIGSNT	SNN	AAWDDSLNGPVV
**PLC8**	IGLV1-51	IGLJ3	SSNIGNNY	DNN	GTWDSSLSAVV
**PLC9**	IGLV3-1	IGLJ3	KLGDKY	QDS	QAWDSSTVV

Supplementary Table 2. List of public light chains screened in this study. Light chain V-genes were derived from a previously published dataset of homeostatic repertoires from three donors. For each of these V-genes, its most commonly associated germline J-gene was chosen.

Supplementary Table 3. Binding dynamics of N3-1 IgG mutants against D614G-Hexapro (WT) and Omicron variants

Kd (M)			Omicron variant			
		D614G	BA.1	BA.2	BA.2.12	BA.5
N3-1 mutant	WT	OR	1.40E-06	8.00E-07	3.27E-07	1.36E-06
	I	OR	3.31E-07	1.21E-07	7.69E-08	1.45E-07
	L	6.00E-09	6.60E-07	2.73E-07	1.73E-07	4.14E-07
	M	4.22E-09	4.28E-07	1.78E-07	2.80E-08	1.72E-07
	Q	OR	4.80E-06	4.83E-07	2.91E-07	7.71E-07
	V	3.78E-09	1.34E-06	7.04E-07	4.21E-07	1.47E-06
	Y	5.45E-09	7.09E-07	2.25E-07	1.42E-07	3.37E-07
**kon (1/Ms)**						
N3-1 mutant	WT	7.07E+04	2.82E+03	6.41E+03	9.40E+03	6.89E+03
	I	7.60E+04	5.82E+03	1.21E+04	1.46E+04	1.16E+04
	L	7.83E+04	6.49E+03	1.52E+04	1.95E+04	1.66E+04
	M	6.59E+04	8.59E+03	1.38E+04	2.22E+04	1.47E+04
	Q	1.00E+03	3.12E+03	8.36E+03	1.15E+04	8.64E+03
	V	6.09E+04	5.41E+03	9.67E+03	1.29E+04	1.11E+04
	Y	1.37E+05	1.12E+04	1.99E+04	2.71E+04	2.13E+04
**koff (1/s)**						
N3-1 mutant	WT	OR	3.93E-03	5.12E-03	3.07E-03	9.40E-03
	I	OR	1.93E-03	1.46E-03	1.12E-03	1.68E-03
	L	4.70E-04	4.29E-03	4.15E-03	3.39E-03	6.89E-03
	M	2.78E-04	3.68E-03	2.45E-03	6.21E-04	2.53E-03
	Q	OR	1.50E-02	4.03E-03	3.36E-03	6.66E-03
	V	2.30E-04	7.26E-03	6.81E-03	5.42E-03	1.63E-02
	Y	7.48E-04	7.91E-03	4.47E-03	3.85E-03	7.20E-03

Supplementary Table 4. Screening of N3-1 IgG double-mutants against D614G and BA1

N3-1 Variant	D614G koff(1/s)	D614G kon(1/Ms)	D614G KD(M)	D614G R2	BA1 koff(1/s)	BA1 kon(1/Ms)	BA1 KD(M)	BA1 R2	Fold change vs wt
0324-LN3_T65H	2.91E-04	1.06E+06	2.76E-10	0.993	4.13E-04	3.56E+05	1.16E-09	0.977	39.91
0305-IN3_T65H	9.99E-05	2.58E+02	3.87E-07	0.98	3.68E-04	1.28E+05	2.89E-09	0.979	16.02
0300-IN3_T65A	OR	2.10E+02	OR	0.973	4.04E-04	1.12E+05	3.61E-09	0.972	12.83
0308-IN3_T65L	1.34E-04	2.61E+02	5.15E-07	0.976	3.64E-04	9.30E+04	3.92E-09	0.983	11.81
0306-IN3_T65I	1.36E-04	2.59E+02	5.24E-07	0.983	5.17E-04	1.19E+05	4.36E-09	0.985	10.62
0303-IN3_T65F	1.15E-04	8.87E+05	1.29E-10	0.974	5.75E-04	9.52E+04	6.04E-09	0.975	7.67
0301-IN3_T65D	6.75E-05	2.21E+02	3.06E-07	0.98	6.01E-04	9.42E+04	6.38E-09	0.968	7.26
0313-IN3_T65R	5.16E-04	5.91E+05	8.73E-10	0.971	7.58E-04	1.03E+05	7.36E-09	0.978	6.29
0342-MN3_T65G	2.17E-04	3.14E+03	6.91E-08	0.987	4.28E-04	5.30E+04	8.08E-09	0.99	5.73
0302-IN3_T65E	1.63E-04	2.10E+02	7.76E-07	0.968	6.34E-04	7.67E+04	8.27E-09	0.98	5.60
0387-YN3_T65P	3.00E-03	2.70E+02	1.11E-05	0.887	9.10E-05	9.88E+03	9.21E-09	0.975	5.03
0316-IN3_T65V	2.62E-05	2.94E+02	8.93E-08	0.985	5.55E-04	5.24E+04	1.06E-08	0.963	4.37
0311-IN3_T65P	5.78E-04	2.89E+03	2.00E-07	0.968	9.65E-04	8.82E+04	1.09E-08	0.968	4.25
0333-LN3_T65S	4.17E-05	2.85E+02	1.46E-07	0.988	5.66E-04	4.85E+04	1.17E-08	0.99	3.96
0337-LN3_T65Y	3.93E-04	4.50E+03	8.73E-08	0.983	3.05E-04	2.61E+04	1.17E-08	0.981	3.96
0370-N3_T65R	3.63E-05	3.28E+02	1.11E-07	0.988	2.66E-04	2.20E+04	1.21E-08	0.965	3.83
0312-IN3_T65Q	5.11E-04	2.29E+05	2.23E-09	0.984	8.87E-04	6.73E+04	1.32E-08	0.99	3.51
0377-YN3_T65D	1.53E-04	2.42E+02	6.34E-07	0.964	2.69E-04	2.01E+04	1.34E-08	0.967	3.46
0310-IN3_T65N	4.48E-04	1.21E+02	3.69E-06	0.957	9.49E-04	6.25E+04	1.52E-08	0.986	3.05
0309-IN3_T65M	OR	1.84E+02	OR	0.949	1.02E-03	6.48E+04	1.57E-08	0.988	2.95
0373-N3_T65V	4.61E-04	1.42E+03	3.24E-07	0.964	5.65E-04	3.52E+04	1.60E-08	0.984	2.89
0317-IN3_T65W	2.82E-04	1.99E+05	1.41E-09	0.991	8.34E-04	5.12E+04	1.63E-08	0.93	2.84
0360-N3_T65F	2.17E-04	6.12E+02	3.55E-07	0.991	6.59E-04	3.57E+04	1.85E-08	0.98	2.50
0315-IN3_T65T	7.96E-04	6.51E+02	1.22E-06	0.95	1.36E-03	6.67E+04	2.04E-08	0.948	2.27
0338-MN3_T65A	1.21E-04	3.25E+02	3.73E-07	0.966	1.54E-03	6.18E+04	2.50E-08	0.989	1.85
0304-IN3_T65G	2.21E-04	4.88E+03	4.54E-08	0.983	6.70E-04	2.39E+04	2.80E-08	0.792	1.65
0339-MN3_T65D	2.00E-05	3.35E+03	5.95E-09	0.963	1.87E-03	6.40E+04	2.92E-08	0.343	1.59
0318-IN3_T65Y	2.22E-04	2.07E+05	1.07E-09	0.995	1.17E-03	3.98E+04	2.94E-08	0.98	1.57
0368-N3_T65P	2.53E-04	4.79E+03	5.29E-08	0.952	3.83E-04	1.28E+04	2.98E-08	0.0333	1.55
0307-IN3_T65K	5.60E-05	2.69E+05	2.08E-10	0.989	8.72E-04	2.75E+04	3.17E-08	0.983	1.46
0330-LN3_T65P	2.19E-03	3.19E+03	6.86E-07	0.939	2.29E-03	7.02E+04	3.26E-08	0.972	1.42
0353-MN3_T65T	1.51E-03	3.42E+02	4.42E-06	0.905	1.05E-03	3.17E+04	3.32E-08	0.981	1.39
0388-YN3_T65Q	1.86E-03	9.73E+02	1.91E-06	0.922	5.80E-04	1.54E+04	3.77E-08	0.972	1.23
0331-LN3_T65Q	8.30E-04	7.03E+05	1.18E-09	0.931	2.23E-03	5.84E+04	3.82E-08	0.985	1.21
0350-MN3_T65Q	1.50E-04	2.33E+02	6.43E-07	0.979	8.71E-04	2.27E+04	3.85E-08	0.965	1.20
0332-LN3_T65R	6.55E-04	3.58E+02	1.83E-06	0.972	1.80E-03	4.62E+04	3.89E-08	0.99	1.19
0347-MN3_T65M	2.33E-04	2.64E+02	8.80E-07	0.977	2.09E-03	5.27E+04	3.97E-08	0.955	1.17
0341-MN3_T65F	1.79E-04	1.23E+03	1.46E-07	0.986	1.71E-03	4.15E+04	4.12E-08	0.972	1.12
0345-MN3_T65K	7.97E-05	2.30E+02	3.46E-07	0.972	1.72E-03	4.16E+04	4.14E-08	0.981	1.12
0374-N3_T65W	OR	1.90E+02	OR	0.972	6.94E-04	1.63E+04	4.25E-08	0.989	1.09
0348-MN3_T65N	9.93E-05	7.65E+02	1.30E-07	0.981	1.96E-03	4.57E+04	4.30E-08	0.992	1.08
N3-1 (WT)	OR	2.99E+02	OR	0.965	4.22E-04	9.11E+03	4.63E-08	0.975	1.00
0340-MN3_T65E	7.27E-05	1.96E+02	3.71E-07	0.966	2.10E-03	4.37E+04	4.82E-08	0.993	0.96
0335-LN3_T65V	5.02E-04	3.73E+03	1.35E-07	0.877	1.96E-03	3.97E+04	4.92E-08	0.974	0.94
0383-YN3_T65K	1.78E-04	2.61E+02	6.82E-07	0.987	9.90E-04	1.96E+04	5.06E-08	0.948	0.92
0327-LN3_T65L	1.27E-03	6.95E+03	1.83E-07	0.889	1.47E-03	2.51E+04	5.86E-08	0.953	0.79
0319-LN3_T65A	8.36E-04	3.43E+03	2.44E-07	0.966	2.29E-03	3.84E+04	5.96E-08	0.925	0.78
0346-MN3_T65L	4.21E-04	1.27E+02	3.32E-06	0.931	2.52E-03	3.92E+04	6.42E-08	0.983	0.72
0386-YN3_T65N	1.38E-03	1.56E+05	8.83E-09	0.981	5.20E-04	7.85E+03	6.63E-08	0.964	0.70
0354-MN3_T65V	1.18E-03	4.03E+03	2.92E-07	0.968	1.53E-03	2.27E+04	6.76E-08	0.987	0.68
0314-IN3_T65S	3.40E-04	7.52E+01	4.52E-06	0.985	1.14E-03	1.66E+04	6.87E-08	0.992	0.67
0358-N3_T65D	OR	1.74E+02	OR	0.934	8.57E-04	1.25E+04	6.88E-08	0.979	0.67
0379-YN3_T65F	2.02E-04	1.01E+02	2.00E-06	0.987	1.05E-03	1.17E+04	8.93E-08	0.976	0.52
0329-LN3_T65N	5.19E-04	2.78E+03	1.87E-07	0.962	9.25E-04	9.38E+03	9.87E-08	0.974	0.47
0371-N3_T65S	1.57E-04	2.65E+02	5.92E-07	0.954	1.13E-03	1.04E+04	1.08E-07	0.981	0.43
0352-MN3_T65S	1.40E-03	2.01E+05	6.97E-09	0.974	1.70E-03	1.37E+04	1.24E-07	0.964	0.37
0344-MN3_T65I	5.00E-04	2.99E+02	1.67E-06	0.979	2.60E-03	1.72E+04	1.51E-07	0.974	0.31
0351-MN3_T65R	8.06E-04	2.08E+05	3.87E-09	0.976	1.87E-03	1.07E+04	1.75E-07	0.965	0.26
0362-N3_T65H	4.60E-04	1.45E+03	3.18E-07	0.952	1.14E-03	3.02E+03	3.78E-07	0.993	0.12
0367-N3_T65N	1.75E-04	1.60E+05	1.09E-09	0.993	1.76E-03	2.74E+03	6.42E-07	0.973	0.07
0359-N3_T65E	2.21E-04	1.81E+05	1.22E-09	0.993	2.69E-04	2.83E+02	9.48E-07	0.966	0.05
0363-N3_T65I	2.30E-05	3.24E+02	7.10E-08	0.983	1.06E-03	6.73E+02	1.58E-06	0.965	0.03
0378-YN3_T65E	1.68E-04	9.06E+05	1.86E-10	0.977	4.39E-04	2.53E+02	1.74E-06	0.985	0.03
0391-YN3_T65T	3.25E-04	1.40E+03	2.33E-07	0.977	2.13E-03	6.00E+02	3.55E-06	0.932	0.01
0380-YN3_T65G	3.11E-04	4.02E+03	7.73E-08	0.978	2.18E-03	4.38E+02	4.98E-06	0.92	0.01
0372-N3_T65T	1.16E-04	2.03E+05	5.70E-10	0.994	1.94E-03	3.00E+02	6.46E-06	0.936	0.01
0381-YN3_T65H	1.60E-04	2.42E+02	6.63E-07	0.98	2.17E-03	2.69E+02	8.04E-06	0.958	0.01
0349-MN3_T65P	1.17E-03	3.92E+05	2.97E-09	0.969	2.19E-03	2.65E+02	8.26E-06	0.986	0.01
0328-LN3_T65M	1.24E-03	4.15E+05	2.99E-09	0.916	3.06E-03	2.71E+02	1.13E-05	0.966	0.00
0334-LN3_T65T	3.70E-04	6.84E+03	5.41E-08	0.976	3.57E-03	2.34E+02	1.52E-05	0.955	0.00
0376-YN3_T65A	4.25E-04	2.33E+05	1.82E-09	0.964	7.98E-04	4.74E+01	1.68E-05	0.982	0.00
0361-N3_T65G	1.77E-04	3.23E+02	5.49E-07	0.978	1.56E-03	9.21E+01	1.70E-05	0.959	0.00
0336-LN3_T65W	1.17E-04	7.82E+05	1.50E-10	0.974	1.37E-03	6.29E+01	2.18E-05	0.949	0.00
0366-N3_T65M	2.08E-06	2.55E+02	8.15E-09	0.971	4.31E-04	1.86E+01	2.31E-05	0.964	0.00
0322-LN3_T65F	7.26E-04	2.88E+05	2.52E-09	0.969	2.43E-03	1.04E+02	2.33E-05	0.99	0.00
0343-MN3_T65H	6.43E-04	8.28E+01	7.76E-06	0.96	2.08E-03	8.24E+01	2.53E-05	0.976	0.00
0384-YN3_T65L	9.57E-04	2.25E+05	4.26E-09	0.968	4.32E-04	1.66E+01	2.61E-05	0.948	0.00
0325-LN3_T65I	1.31E-03	1.99E+05	6.57E-09	0.907	6.32E-04	2.32E+01	2.72E-05	0.979	0.00
0393-YN3_T65W	5.35E-04	2.84E+03	1.89E-07	0.979	7.50E-04	1.68E+01	4.46E-05	0.957	0.00
0355-MN3_T65W	4.10E-04	8.65E+05	4.74E-10	0.976	2.06E-03	4.51E+01	4.57E-05	0.988	0.00
0323-LN3_T65G	9.32E-04	6.42E+03	1.45E-07	0.984	1.99E-03	4.20E+01	4.74E-05	0.927	0.00
0320-LN3_T65D	7.16E-04	3.16E+03	2.26E-07	0.965	3.01E-03	6.27E+01	4.81E-05	0.937	0.00
0357-N3_T65A	1.24E-04	2.21E+05	5.62E-10	0.991	1.90E-03	3.43E+01	5.54E-05	0.412	0.00
0375-N3_T65Y	OR	2.65E+02	OR	0.987	1.54E-03	2.57E+01	5.97E-05	0.984	0.00
0356-MN3_T65Y	4.94E-03	1.23E+02	4.01E-05	0.752	2.18E-03	3.55E+01	6.14E-05	0.972	0.00
0321-LN3_T65E	1.57E-03	4.15E+03	3.78E-07	0.903	2.53E-03	4.02E+01	6.30E-05	0.94	0.00
0365-N3_T65L	1.23E-04	3.18E+02	3.87E-07	0.988	2.20E-03	3.39E+01	6.48E-05	0.987	0.00
0394-YN3_T65Y	4.12E-04	7.27E+02	5.66E-07	0.809	8.46E-04	1.27E+01	6.67E-05	0.967	0.00
0392-YN3_T65V	4.75E-03	2.65E+02	1.79E-05	0.872	9.77E-04	1.38E+01	7.07E-05	0.978	0.00
0389-YN3_T65R	1.07E-03	1.28E+03	8.39E-07	0.923	2.12E-03	2.49E+01	8.48E-05	0.943	0.00
0390-YN3_T65S	1.05E-03	1.27E+03	8.30E-07	0.891	2.29E-03	2.14E+01	1.07E-04	0.903	0.00
0382-YN3_T65I	8.11E-04	3.02E+02	2.69E-06	0.955	2.94E-03	1.65E+01	1.78E-04	0.965	0.00
0364-N3_T65K	9.92E-05	3.44E+02	2.88E-07	0.988	2.37E-03	1.16E+01	2.03E-04	0.942	0.00
0326-LN3_T65K	1.07E-03	2.94E+02	3.65E-06	0.967	4.13E-04	OR	OR	0.926	-
0369-N3_T65Q	1.13E-04	1.73E+03	6.54E-08	0.969	1.96E-03	OR	OR	0.914	-
0385-YN3_T65M	1.57E-03	1.58E+05	9.93E-09	0.978	OR	4.16E+01	OR	0.982	-
