# Supplementary material for: SARS-COV-2 Omicron variants conformationally escape a rare quaternary antibody binding mode
Source: Commun Biol. 2023 Dec 11;6:1250. doi: 10.1038/s42003-023-05649-6 (PMC10713552; doi:10.1038/s42003-023-05649-6)
Supplement: Supplementary file 5 — Reporting Summary [file 42003_2023_5649_MOESM5_ESM.pdf]

nature portfolio

Corresponding author(s): Daniel R. Boutz, Jason S. McLellan, Jimmy D. Gollihar

Last updated by author(s): Aug 29, 2023

Reporting Summary

Nature Portfolio wishes to improve the reproducibility of the work that we publish. This form provides structure for consistency and transparency in reporting. For further information on Nature Portfolio policies, see our Editorial Policies and the Editorial Policy Checklist.

Statistics

For all statistical analyses, confirm that the following items are present in the figure legend, table legend, main text, or Methods section.

n/a	Confirmed	
☐	☒	The exact sample size (*n*) for each experimental group/condition, given as a discrete number and unit of measurement
☐	☒	A statement on whether measurements were taken from distinct samples or whether the same sample was measured repeatedly
☐	☒	The statistical test(s) used AND whether they are one- or two-sided<br>*Only common tests should be described solely by name; describe more complex techniques in the Methods section.*
☒	☐	A description of all covariates tested
☒	☐	A description of any assumptions or corrections, such as tests of normality and adjustment for multiple comparisons
☒	☐	A full description of the statistical parameters including central tendency (e.g. means) or other basic estimates (e.g. regression coefficient) AND variation (e.g. standard deviation) or associated estimates of uncertainty (e.g. confidence intervals)
☒	☐	For null hypothesis testing, the test statistic (e.g. *F*, *t*, *r*) with confidence intervals, effect sizes, degrees of freedom and *P* value noted<br>*Give P values as exact values whenever suitable.*
☒	☐	For Bayesian analysis, information on the choice of priors and Markov chain Monte Carlo settings
☒	☐	For hierarchical and complex designs, identification of the appropriate level for tests and full reporting of outcomes
☒	☐	Estimates of effect sizes (e.g. Cohen's *d*, Pearson's *r*), indicating how they were calculated

Our web collection on statistics for biologists contains articles on many of the points above.

Software and code

Policy information about availability of computer code

Data collection	Xcalibur (Thermo Scientific); MinKNOW (Oxford Nanopore); GatorOne 2.10.0713 (GatorBio); SA3900 Spectral Analyzer software (Sony); ASTRA 8.2.0 (Wyatt Technology Corp)
Data analysis	Proteome Discoverer 2.2 (Thermo Scientific); Guppy 3.5.2 (Oxford Nanopore); Guppy 4.0.11 (Oxford Nanopore); MiXCR v3.0.13; minimap2 2.17; Racon 1.4.13 (Oxford Nanopore); kalign 3.2.3; in-house nanopore analysis scripts (described in Methods and Fig S6); FlowJo v9 (BD Bioscience); GatorOne 2.10.0713 (GatorBio); Harmony (PerkinElmer); Prism 9 (GraphPad); Biacore X100 Evaluation Software (GE Healthcare); cisTEM; DeepEMhancer; ChimeraX (UCSF); Warp; cryoSPARC v2.15.0; cryoSPARC v4.2.1; Coot; PHENIX v1.20.1; ISOLDE; PISA; EMDB validation analysis 0.0.1.dev50; wwPDB-VP 2.35; ASTRA 8.2.0 (Wyatt Technology Corp)

For manuscripts utilizing custom algorithms or software that are central to the research but not yet described in published literature, software must be made available to editors and reviewers. We strongly encourage code deposition in a community repository (e.g. GitHub). See the Nature Portfolio guidelines for submitting code & software for further information.

Data

Policy information about availability of data

All manuscripts must include a data availability statement. This statement should provide the following information, where applicable:

- Accession codes, unique identifiers, or web links for publicly available datasets
- A description of any restrictions on data availability
- For clinical datasets or third party data, please ensure that the statement adheres to our policy

Molecular coordinates for N3-1 Fab complexes with SARS-CoV-2 RBDs have been deposited to the Protein Data Bank (PDB ID: 8TM1 and 8TMA). One global EM map (EMD-41399) and two focused refinement maps (EMD-41374 and EMD-41382) are available at the Electron Microscopy Data Bank. Structural data are presented in Fig. 3, Supplementary Table 5, and Supplementary Figs. 8 and 15-16.

Research involving human participants, their data, or biological material

Policy information about studies with human participants or human data. See also policy information about sex, gender (identity/presentation), and sexual orientation and race, ethnicity and racism.

Reporting on sex and gender	N/A
Reporting on race, ethnicity, or other socially relevant groupings	N/A
Population characteristics	Blood was collected from two PCR-confirmed patients at day 12 post-onset of symptoms. Neither donor was hospitalized or experienced severe disease. Donors 1 and 2 were both female, aged 65 and 35 respectively.
Recruitment	Donors were identified among the first SARS-CoV-2 wave in central Texas and responded positively to our inquiry.
Ethics oversight	The acquisition of blood specimens from convalescent individuals was approved by the University of Texas at Austin Institutional Review Board (protocol 2020-03-085; Breadth of serum antibody immune responses prior to, or following, patient recovery in asymptomatic and non-severe COVID-19).

Note that full information on the approval of the study protocol must also be provided in the manuscript.

Field-specific reporting

Please select the one below that is the best fit for your research. If you are not sure, read the appropriate sections before making your selection.

☒ Life sciences ☐ Behavioural & social sciences ☐ Ecological, evolutionary & environmental sciences

For a reference copy of the document with all sections, see nature.com/documents/nr-reporting-summary-flat.pdf

Life sciences study design

All studies must disclose on these points even when the disclosure is negative.

Sample size	N/A. We draw no inferences that depend on number of donors recruited.
Data exclusions	No data was excluded from this study.
Replication	We performed mAb characterizations on biological duplicates or triplicates. Neutralization assays were performed by three independent laboratories each at a different institution.
Randomization	N/A. We do not compare between different groups.
Blinding	N/A. We do not compare between different groups.

Reporting for specific materials, systems and methods

We require information from authors about some types of materials, experimental systems and methods used in many studies. Here, indicate whether each material, system or method listed is relevant to your study. If you are not sure if a list item applies to your research, read the appropriate section before selecting a response.

Materials & experimental systems

n/a	Involved in the study
☐	☒ Antibodies
☐	☒ Eukaryotic cell lines
☒	☐ Palaeontology and archaeology
☒	☐ Animals and other organisms
☒	☐ Clinical data
☒	☐ Dual use research of concern
☒	☐ Plants

Methods

n/a	Involved in the study
☒	☐ ChIP-seq
☐	☒ Flow cytometry
☒	☐ MRI-based neuroimaging

Antibodies

Antibodies used

Mouse anti-FLAG M2 Sigma-Aldrich Cat# F3165; RRID:AB_259529
Goat anti-Mouse IgG(H+L), Human ads-Alexa Fluor 488 Southern Biotech Cat# 1031-30; RRID:AB_2794315
Goat anti-Human IgG Fc-Alexa Fluor 647 Southern Biotech Cat# 2048-31; RRID:AB_2795692
secondary goat anti-human Fab-HRP (Sigma-Aldrich, cat. no. A0293)

Validation

All antibodies were assayed using standard controls upon receipt from the manufacturer.

1. Mouse anti-FLAG M2 (Sigma-Aldrich)
Catalog #: F3165
RRID: AB_259529

Validation:
The Mouse anti-FLAG M2 antibody has been rigorously tested for specificity and sensitivity. This antibody specifically binds to the FLAG epitope in both Western blot and immunoprecipitation applications. Additionally, the lack of binding in FLAG-negative controls further validates its specificity.

2. Goat anti-Mouse IgG(H+L), Human ads-Alexa Fluor 488 (Southern Biotech)
Catalog #: 1031-30
RRID: AB_2794315

Validation:
The Goat anti-Mouse IgG(H+L) conjugated to Alexa Fluor 488 has been thoroughly validated to ensure optimal performance. It exhibits strong fluorescence when binding to mouse IgG and negligible cross-reactivity with other species, ensuring specificity. This secondary antibody was also validated against human immunoglobulins.

3. Goat anti-Human IgG Fc-Alexa Fluor 647 (Southern Biotech)
Catalog #: 2048-31
RRID: AB_2795692

Validation:
The Goat anti-Human IgG Fc antibody labeled with Alexa Fluor 647 has been validated in various applications including flow cytometry and immunofluorescence. This secondary antibody shows a high degree of specificity for the Fc region of human IgG without any notable binding to the Fab region. When tested in samples without human IgG, there was minimal to no background fluorescence.

4. Secondary Goat anti-Human Fab-HRP (Sigma-Aldrich)
Catalog #: A0293

Validation:
The Goat anti-Human Fab-HRP secondary antibody has undergone rigorous testing for specificity and sensitivity in applications like ELISA and Western blotting. It demonstrates a high specificity for the Fab region of human IgG antibodies and does not exhibit any cross-reactivity with the Fc region. The conjugated horseradish peroxidase (HRP) activity is consistent across batches, ensuring reproducibility.

Eukaryotic cell lines

Policy information about cell lines and Sex and Gender in Research

Cell line source(s)

HEK293T cells (female) were acquired from ATCC (cat. no. CRL-3216).
Expi293F cells (female) were acquired from Thermo Fisher (cat. no. A14527).
Vero E6 cells (female) were acquired from ATCC (cat. no. CRL-1586).

Authentication

Cell lines for production of recombinant proteins were not authenticated prior to use.

Mycoplasma contamination

Cells lines were tested for mycoplasma contamination before use via the Mycoplasma Detection Kit (SouthernBiotech 13100-01).

Commonly misidentified lines (See ICLAC register)	N/A

Flow Cytometry

Plots

Confirm that:

☒ The axis labels state the marker and fluorochrome used (e.g. CD4-FITC).

☒ The axis scales are clearly visible. Include numbers along axes only for bottom left plot of group (a 'group' is an analysis of identical markers).

☒ All plots are contour plots with outliers or pseudocolor plots.

☒ A numerical value for number of cells or percentage (with statistics) is provided.

Methodology

Sample preparation	EBY100 or HEK293T cells were grown in rich media prior to sorting or cytometry. Pelleted cells were washed before primary staining and again after secondary labeling.
Instrument	Sony SA3800 Spectral Cell Analyzer<br>Sony SH800 fluorescent cell sorter
Software	FlowJo v9 (BD Bioscience)
Cell population abundance	Sorted cells were grown and sequenced between rounds. We aimed to collect 0.5% of cells within each gate. Individual clones were validated by sequencing.
Gating strategy	Gates were drawn to collect high expression or high binding populations. We used unlabeled and single-label controls to determine range and unmixing.

☒ Tick this box to confirm that a figure exemplifying the gating strategy is provided in the Supplementary Information.
